# Visualization of Airborne Particles as a Risk for Microbial Contamination in Orthopedic Surgery

**DOI:** 10.3389/fsurg.2021.754785

**Published:** 2021-11-22

**Authors:** Hironobu Koseki, Shinya Sunagawa, Chieko Imai, Akihiko Yonekura, Umi Matsumura, Seiichi Yokoo, Kaho Watanabe, Yuta Nishiyama, Makoto Osaki

**Affiliations:** ^1^Departments of Health Sciences, Nagasaki University Graduate School of Biomedical Sciences, Nagasaki, Japan; ^2^Department of Rehabilitation, Wajinkai Hospital, Nagasaki, Japan; ^3^Departments of Orthopedic Surgery, Nagasaki University Graduate School of Biomedical Sciences, Nagasaki, Japan; ^4^Departments of Nursing, Fukuoka International University of Health and Welfare, Fukuoka, Japan

**Keywords:** surgery, airborne particles, microbial contamination, occupational infection, orthopedic surgery

## Abstract

**Background:** The operating theater is recognized to involve a high frequency of occupational blood and body fluid contacts.

**Objectives:** This study aimed to visualize the production of blood and body fluid airborne particles by surgical procedures and to investigate risks of microbial contamination of the conjunctival membranes of surgical staff during orthopedic operations.

**Methods:** Two physicians simulated total knee arthroplasty (TKA) and total hip arthroplasty (THA) in a bio-clean theater using model bones. The generation and behaviors of airborne particles were filmed using a fine particle visualization system, and numbers of airborne particles per 2.83 L of air were counted at the height of the operating and instrument tables. Each action was repeated five times, and particle counts were evaluated statistically.

**Results:** Numerous airborne particles were dispersed to higher and wider areas while “cutting bones in TKA” and “striking and driving the cup component on the pelvic bone in THA” compared to other surgical procedures. The highest particle counts were detected while “cutting bones in TKA” under unidirectional laminar air flow.

**Discussion:** These results provide a clearer image of the dispersion and distribution of airborne particles and identified higher-risk surgical procedures for microbial contamination of the conjunctival membranes. Surgical staff including surgeons, nurses, anesthesiologists, and visitors, should pay attention to and take measures against occupational infection particularly in high-risk surgical situations.

## Introduction

Despite improvements in preventive strategies, healthcare workers (HCWs) still face the risk of bloodborne infection from occupational exposures to human blood and body fluids (BBF) (1, 2). BBF can lead to transmission of fatal infectious diseases, such as hepatitis B (HBV), hepatitis C (HCV) and human immunodeficiency virus (HIV), some with unfavorable prognoses (3, 4). The incidences of occupational transmission of HBV, HCV, and HIV to HCWs after percutaneous exposure from an infected source patient rest at about 30.0%, 3.0%, and 0.09–0.3%, respectively (5, 6). Pathogens can be transmitted to HCWs through sharps injuries (needlestick or cut by a scalpel blade or any other sharp object contaminated with BBF from an infected patient) or mucocutaneous exposure (MCE; contact of BBF with non-intact skin or the mucosa of the eyes, nose or mouth). In Japan, a nationwide epidemiological study has been conducted annually since 1996, and reported 7,397 cases of bloodborne exposure in 93 key hospitals between 2013 and 2014 (7). The research revealed that these exposures comprised 6,201 sharps injuries (83.8%) and 1,196 MCEs (16.2%), with occupational BBF exposures occurring more frequently among nurses (49%) than among doctors (36%). Although most previous studies have focused on sharps injuries (8, 9), the annual Exposure Survey of Trends in Occupational Practice (EXPO-S.T.O.P) in 2017, including 33 states in the United States, reported MCEs occurring in 0.87% of full-time equivalent staff and the incident rate had increased over the 2011 rate in all hospital categories (10).

The operating theater is recognized as showing a high frequency of occupational BBF contact because of prolonged contact of surgical personnel with open surgical sites, frequent manipulation of sharp instruments, and the presence of relatively large quantities of BBF (4, 11–14). Endo et al. (15) evaluated facial blood spatter rates and locations on 600 face masks of surgical staff, including lead surgeons, first assistants, and scrub nurses. They revealed that 74.4% (447/600) of blood spatters were detected in the orbital or para-orbital regions, with an incidence of 60.0% (90/150) in orthopedic surgeries. Biological and epidemiological evidence suggests that the deposition of a sufficient number of infective BBF particles on the mucous membranes can lead to HCV or HIV-1 transmission (5, 16). In the orthopedic field, total joint arthroplasties are thought to generate large quantities of aerosol particles and bone dust in the air (17, 18). However, surgeons are often unaware of potentially infectious BBF spatter, as their attention is focused on the surgical procedures being performed (14, 17, 19). Awareness of exposure to blood spatter among surgeons was 8.0% according to Marasco and Woods (14) and 0% according to Keogh et al. (19). Surgical staff should thus pay attention to protecting the face and conjunctival membranes of surrounding surgical staff against exposure to potentially infective surgical debris. However, the frequency of using facial shields has been reported to be as low as 4.0% (3), because upper facial protection is uncomfortable and obstructs vision or because staff lack awareness of the need to protect against intraoperative BBF splashes (18, 19). As the dispersal and flow of BBF particles during surgery has not been visualized to date, the mechanisms and degree to which they are generated by surgical procedures remain unclear. Each staff member in an operating theater needs to understand which situations are risky and increase the number of BBF particles.

The present study aimed to quantify the dispersion and distribution of airborne particles of BBF arising in two major orthopedic surgeries, to evoke awareness in surgical staff about MCE and to help reduce the risks of occupational infection.

## Materials and Methods

### Experimental Design

Two orthopedic physicians were recruited for the present study. After donning a surgical cap and mask, each participant put on a surgical gown (JG-100^®^ Hopes Co., Hokkaido, Japan) and latex powdered surgical gloves (Tradition^®^ Medline International Japan, Tokyo, Japan). All surgical garments used in this study were made from standard spun-lace non-woven fabric comprising 45% wood pulp and 55% polyester pulp. The physicians performed two types of simulated surgery on an operating table (height, 1.1 m; width, 50 cm; length, 212 cm) located in the center of a unidirectional laminar airflow (LAF)-equipped bio-clean theater, which creates a homogeneous, low-turbulence vertical airflow directly over the operating area through a combination of high airflow rates and high-efficacy particulate air (HEPA) filtration (20). The bio-clean theater was an ISO class 7, Fed. Standard class 10,000 and LAF settings were as follows: air velocity, 0.44 m/s; theater temperature, 21.9°C; humidity, 32.4%; and air pressure, 15 Pa (1.53 mmAq) positive (21).

The simulated surgeries comprised total knee arthroplasty (TKA) and total hip arthroplasty (THA) using sawbones models in actual situations. TKA was performed with an anterior-cut-first and anterior reference technique and a non-cement implantation method with cruciate-retaining total knee components (Genesis II^®^ Smith & Nephew Orthopedics, Memphis, TN). Distilled water to mimic blood was soaked in sawbones (SKU:1179; Pacific Research Laboratories, Vashon, WA, USA) and the bone models were fixed to the metal flame on the operating table. The distal femoral condyle and proximal part of the tibia were resected and molded procedurally using appropriate cutting blocks and a bone saw. After prosthetic trialing, femoral and tibial components and articular insert were implanted. THA was performed assuming a posterior approach with non-cement total hip components (Reflection^®^ and Synergy Select II^®^ Smith & Nephew Orthopedics). Distilled water to mimic blood was soaked into sawbones (SKU:1301-170; Pacific Research Laboratories) and the bone models were fixed to an original metal frame on the operating table. After cutting the femoral neck and removing the femoral head, curettage and reaming of the acetabulum were performed in a sequential manner. The medullary cavity of the femur was reamed and broached, then a prosthesis of optimal size was implanted.

The generation and behaviors of airborne particles were filmed using a fine particle visualization system (ViEST^TM^; Shin-Nihon Air Technologies Co., Tokyo, Japan) with a green laser (22, 23). After generating a uniform laser light sheet, light reflected from airborne particles was filmed using a highly sensitive camera with an interference filter. Movies were converted into 1/30-s images, and particle density hazard maps were created using MATLAB^®^ image analysis software (MathWorks Co., Tokyo, Japan). Risk areas were classified as ultra-high (red), high (light blue) or medium (white) zones. The number of airborne particles was determined using a KC-52^®^ laser particle counters (RION, Tokyo, Japan) located 20 cm from both the operating table and instrument table. Samples were collected at 1.1 m above the floor to simulate the height of the operating or instrument tables. A sampling tube (internal diameter, 6 mm) was attached to the air intake port of the particle counter, and the measurement interval was set as 1 min (2.83 L of air per minute), with the mean value used for analysis. Particles were separated based on size distributions of 0.3–0.5, 0.6–1.0, 1.1–2.0, and 2.1–5.0 μm. The theater used in this study was cleaned every morning and evening, and the floor was wiped before each performance. The particle counter in the empty theater constantly showed 50–200 particles/min, and no operating actions were performed until particles had finished dispersing (particle count <200 particles/min).

### Statistical Analysis

Each surgical procedure task was repeated five times. Particle counts were compiled for statistical analysis, which consisted of one-way analysis of variance multiple comparison tests followed by *post hoc* Tukey-Kramer and Bonferroni-Dunn multiple comparison tests. All data were analyzed using SPSS version 22.0 software (SPSS, Chicago, IL, USA). Values are expressed as mean ± standard deviation. Statistical significance was defined for values of *P* < 0.01.

## Results

**Figure 1:**
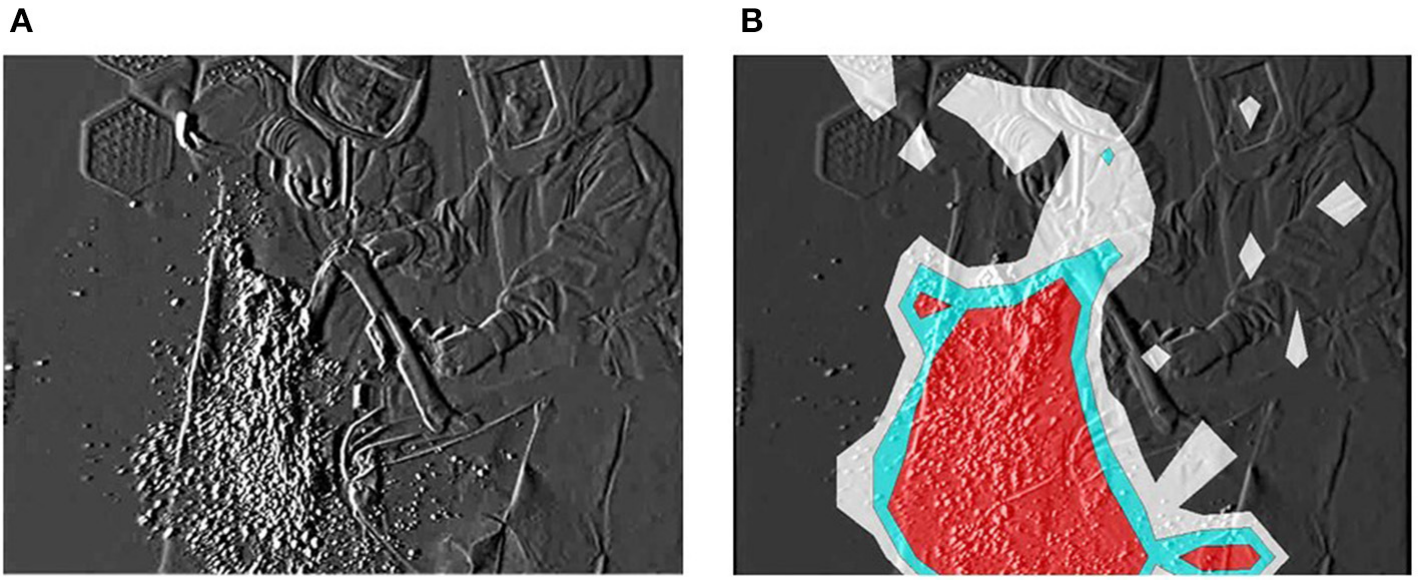
Resecting the distal femoral condyle with the bone saw. The fine particle visualization system shows dispersal of reflective airborne particles (bright dots). **(A)** Many particles including bone tissue and BBF disperse in the direction of saw blade oscillation. **(B)** The particle density hazard map shows risk areas as ultra-high (red), high (light blue) or medium (white) density zones.

**Figure 2:**
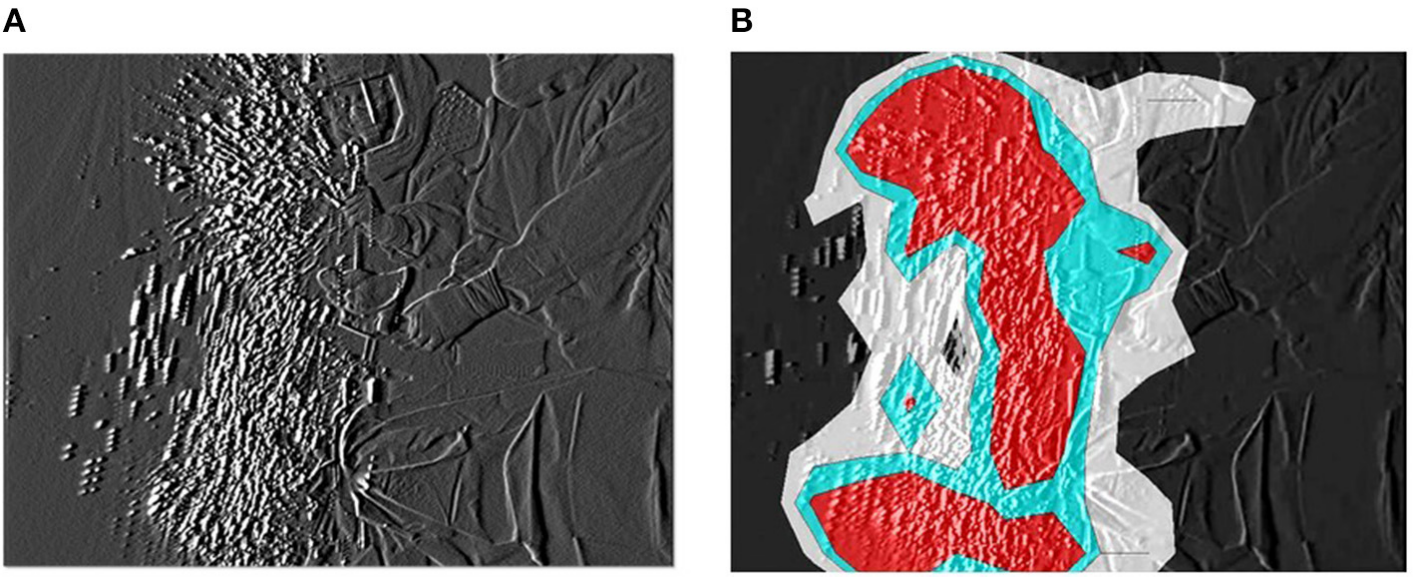
Hammering the trial shell to the acetabulum. **(A)** The generated particles are widely dispersed and carried higher from the acetabulum and drop down to the floor. **(B)** The particle density hazard map displays risk areas expanding in all directions over a 3-m distance.

The dispersal of reflective airborne particles was evidenced by small, bright dots through the fine particle visualization system. In the absence of action, airborne particles in the bio-clean theater drifted down under the LAF and slowly moved away from the center to the perimeter of the theater. Fine particles dispersed around the surgical field throughout TKA and THA processes. In TKA, many particles were dispersed in the direction of blade movement during resection of the femur and tibia from the contact between saw blade and bone (Figure 1A). After turning off the bone saw, most distributed particles hovered for a short period in the air, followed by slow migration down under the LAF. The hazard map constructed during bone resection indicated a high-risk zone spreading in the same direction as the saw blade oscillation (Figure 1B). In THA process, a notable number of particles containing moisture was observed while “hammering the trial shell and impacting the acetabular cup,” and “broaching the femur and implanting the stem into the medullary cavity of the femur” (Figure 2A). Fine particles dispersed from the acetabulum and medullary cavity of the femur and promptly dropped down toward the floor. Hazard maps of “hammering the trial shell to the acetabulum” showed that the high-density area expanded higher and wider compared to other processes in all directions over a 3-m distance (Figure 2B). Table 1 shows the mean number of airborne particles measured during surgical procedures, near both the operating and instrument tables. More particles were detected on the side of the operating table than on the side of the instrument table in all size categories, especially those 0.3–1.0 μm in size (*P* < 0.01).

**Table 1:** Mean number and standard deviation of airborne particles (particles/2.83 L).

**Particle size category (μm)**		**0.3–0.5**	**0.6–1.0**	**1.1–2.0**	**2.1–5.0**	**Total**
TKA femur resection	Operating table	3691 ± 256.3*^§#^	444 ± 13.6*^§^	115 ± 22.3*	180 ± 38.1*	4431 ± 217.1*^§#^
	Instrument table	1030 ± 140.3^§#^	150 ± 29.6	65 ± 18.9	96 ± 31.8	1341 ± 181.4^§#^
THA femur broaching	Operating table	2355 ± 396.7*	272 ± 36.1*	84 ± 10.6*	120 ± 18.1*	2831 ± 450.7*
	Instrument table	807 ± 96.2	110 ± 20.1	28 ± 7.0	35 ± 10.9	980 ± 125.7
THA acetabular hammering	Operating table	2154 ± 407.0*	383 ± 66.9*	89 ± 13.5*	106 ± 14.7*	2732 ± 490.5*
	Instrument table	830 ± 193.6	124 ± 54.7	37 ± 6.7	57 ± 16.6	1047 ± 262.2

* *P < 0.01 compared with instrument table*.

§ *P < 0.01 compared with broaching of the femur in THA*.

# *P < 0.01 compared with acetabular hammering in THA*.

## Discussion

At least 60 bloodborne pathogens can be acquired *via* BBF exposure (2). Although not as lethal as acquired immunodeficiency syndrome, an estimated 12,000 HCWs each year develop hepatitis, resulting in 200–300 deaths (24, 25). Awareness is increasing that surgical staff are also at risk of transmission of bloodborne pathogens from patients through exposure to BBF during operations (4, 11–13, 15). A multicenter national survey in Italy reported accidental exposure to BBF in 9.2% of the total of 15,375 surgeries, with 3.5% and 7.1% as conjunctival and facial exposures, respectively (4). However, upper facial protection such as protective eyewear or face shields is not widely used due to discomfort (17). As reported by Jovic-Vranes et al. (3), facial exposures accounted for 34.4% of BBF exposure among HCWs, but protective facial equipment was utilized in only 3.7%. According to the International Health Care Worker Safety Center (26) based on data from 70 hospitals in the United States, the use of personal protective equipment (PPE) ranged from 1.5% to 59.5% depending on the particular equipment. Furthermore, even with the adoption of preventive strategies, MCEs continue to develop at a constant rate. To reduce the occupational risk of infection for surgical staff, another new approach is desired to deepen the understanding of high-risk surgical procedures for conjunctival contamination and bloodborne infection.

The particle visualization system revealed that airborne particles of BBF continued to disperse around the operating table during TKA and THA. In the United States alone, 719,000 TKAs and 332,000 THAs were carried out in 2010 (27) and numbers of TKAs and THAs are projected to reach 3.48 million and 572,000 by 2030, respectively (28). Many precautions, such as sterile operating environments, peri-operative antibiotics, and antibiotic-loaded bone cement, are applied to avoid surgical site infections for patients. However, no guidelines have been established to avoid MCEs in surgical staff. Singh et al. (18) investigated 110 sets of face and eye PPE used in 55 total joint arthroplasties and revealed that all equipment was contaminated with blood or fat droplets. The video recordings in the present study revealed that the step showing the greatest dispersal of fine airborne particles was resection of the femur and tibia bone in TKA, arising from the tip of saw blade. Dispersed particles were thought to include BBF and crushed bone fragments. Hazard mapping elucidated a high-risk area spreading in the direction of blade movement and a proportion of particles hovered for a short period in the air. LAF with HEPA filters can remove ~99.97% of airborne particles larger than 0.3 μm, resulting in minimal bacterial counts in the air (29, 30). However, the particle counter indicated that the mean number of particles produced by all TKA procedures remained high, particularly during the use of bone saw as a result even under LAF. Floating fine particles can act as vectors for transmission of microorganisms (31–33). Hansen et al. (20) noted that bacterial counts were lower in environments with fewer airborne particles, and that the number of particles ≥5 μm in diameter closely correlated with bacterial concentrations (*P* ≤ 0.01, Spearman correlation). Surgical staff, including surgeons and nurses, should thus pay attention during the use of power tools, especially bone saws, to avoid inhaling particles into the body and to protect the eye and conjunctival membranes from microbial contamination. In THA, meanwhile, a large number of moistened particles were generated and distributed around at high speed while “hammering the trial shell and impacting the acetabular cup” and “broaching the femur and implanting the stem into the medullary cavity of the femur”. Excavated acetabulum and femoral bone marrow tend to accumulate and store blood or body fluids of the patient. Therefore, the impaction procedures of trial, broach, and implantation or insertion of prosthetic components into the acetabulum and medullary cavity of the femur can scatter BBF over large distances. Particles were detected not only on the side of the operating table, but also on the side of the instrument table for particles of all size categories. These procedures may thus increase the potential for facial (conjunctival) contamination of nearby surgical staff, including surgeons, nurses, anesthesiologists, vendors, and visitors. Surgical staff should re-recognize the risk of conjunctival contamination leading to devastating infections, and need to protect their own conjunctival membranes with eyewear or a face shield more frequently and more effectively. Approaches to minimize the risk of occupational infection by MCE have included intelligible evidence for education in PPE usage as well as training in safe surgical techniques and legislative action. The video obtained in the present study suggested that the use of power tools, especially bone saws and impaction tools in total arthroplasty resulted in dispersal of a large number of BBF particles. Surgical staff must pay attention to avoid contamination of their own face and conjunctival membranes, particularly during these high-risk procedures.

Several potential limitations to this study should be noted. We were able to visualize the dispersal of BBF particles and clarify risky procedures for conjunctival contamination of surgical staff during TKA and THA surgeries. However, the simulated surgeries could not completely reproduce the actual surgical situations, which involve additional soft tissues and bleeding in the surgical wound. Further studies simulating various other experimental situations are therefore necessary. In addition, we were unable to assess the actual relationship between particle counts and occupational infection rates. However, more particles can increase the possibility of conjunctival contamination, and awareness of the production and behavior of BBF particles and effective usage of PPE during total arthroplasty is therefore important.

This study provides surgical staff with a clearer image of the dispersal and distribution of BBF particles that may contaminate the conjunctival membranes during several patterns of operating activities within the bio-clean operating theater. To reduce the risk of occupational infection by airborne microorganisms, surgeons should take measures to minimize the production of BBF particles and surgical staff must pay attention to protecting themselves from MCEs.

## Conclusions

Fine particle visualization and automatic particle counting revealed large numbers of BBF particles dispersed and distributed during the use of bone saws and impaction tools in TKA and THA procedures. Surgical staff should re-recognize the risk of occupational infection *via* conjunctival contamination and take measures to minimize opportunities for infected BBF splash onto the conjunctival membranes.

## Data Availability Statement

The datasets presented in this article are not readily available because the dataset is part of ongoing study protocols. Requests to access the datasets should be directed to Hironobu Koseki, koseki@nagasaki-u.ac.jp.

## Ethics Statement

The Ethics Committee at the Nagasaki University Graduate School of Biomedical Sciences granted an exemption from ethics approval because no humans and no human materials were the subjects of this study, and data were not derived from patients.

## Author Contributions

HK conceived and designed the study. SS, CI, UM, SY, YN, and KW participated in the experiments and gathered the data. CI, AY, and MO analyzed and interpreted the data. HK wrote the initial drafts of the manuscript. AY and MO statistically analyzed and ensured the accuracy of the data. All authors have read and approved the final version of the manuscript and affirm that the work has not been submitted or published elsewhere in whole or in part. All authors substantially contributed to this article.

## Funding

This work was partially supported by Grants-in-Aid for Scientific Research from the Japan Society for the Promotion of Science, Grant Number 232024000.

## Conflict of Interest

The authors declare that the research was conducted in the absence of any commercial or financial relationships that could be construed as a potential conflict of interest.

## Publisher's Note

All claims expressed in this article are solely those of the authors and do not necessarily represent those of their affiliated organizations, or those of the publisher, the editors and the reviewers. Any product that may be evaluated in this article, or claim that may be made by its manufacturer, is not guaranteed or endorsed by the publisher.

## References

[1] Beltrami EM Williams IT Shapiro CN Chamberland ME. Risk and management of blood-borne infections in health care workers. Clin Microbiol Rev. (2000) 13:385–407. 10.1128/CMR.13.3.385 10885983 PMC88939

[2] Tarantola A Abiteboul D Rachline A. Infection risks following accidental exposure to blood or body fluids in health care workers: a review of pathogens transmitted in published cases. Am J Infect Control. (2006) 34:367–75. 10.1016/j.ajic.2004.11.011 16877106 PMC7115312

[3] Jovic-Vranes A Jankovic S Vranes B. Safety practice and professional exposure to blood and blood-containing materials in serbian health care workers. J Occup Health. (2006) 48:377–82. 10.1539/joh.48.377 17053304

[4] Pietrabissa A Merigliano S Montorsi M Poggioli G Stella M Borzomati D. Reducing the occupational risk of infections for the surgeon: multicentric national survey on more than 15,000 surgical procedures. World J Surg. (1997) 21:573–8. 10.1007/s002689900275 9230652

[5] Greene DL Akelman E. A technique for reducing splash exposure during pulsatile lavage. J Orthop Trauma. (2004) 18:41–2. 10.1097/00005131-200401000-00008 14676556

[6] Trim JC Elliott TSJ. A review of sharps injuries and preventative strategies. J Hosp Infect. (2003) 53:237–42. 10.1053/jhin.2002.1378 12660120

[7] Research Group of Occupational Infection Control and Prevention in Japan. Japan-EPINet Sharp Object Injury and Blood and Body Fluid Data Reports in Japan (2004–2017). (2021). Available online at: http://jrgoicp.umin.ac.jp/index_jes_reports.html

[8] Kunishima H Yoshida E Caputo J Mikamo H. Estimating the national cost burden of in-hospital needlestick injuries among healthcare workers in Japan. PLoS ONE. (2019) 14:e0224142. 10.1371/journal.pone.0224142 31697746 PMC6837393

[9] Yoshikawa T Wada K Lee JJ Mitsuda T Kidouchi K Kurosu H. Incidence rate of needlestick and sharps injuries in 67 Japanese hospitals: a national surveillance study. PLoS ONE. (2013) 8:e77524. 10.1371/journal.pone.0077524 24204856 PMC3813677

[10] Grimmond T Good L. EXPO-S.T.O.P. 2016 and 2017 blood exposure surveys: An alarming rise. Am J Infect Control. (2019) 47:1465–70. 10.1016/j.ajic.2019.07.004 31402068

[11] Adesunkanmi AK Badmus TA Ogunlusi JO. Accidental injuries and cutaneous contaminations during general surgical operations in a Nigerian teaching hospital. East Afr Med J. (2003) 80:227–34. 10.4314/eamj.v80i5.8691 16167737

[12] Dement JM Epling C Ostbye T Pompeii LA Hunt DL. Blood and body fluid exposure risks among health care workers: results from the Duke Health and Safety Surveillance System. Am J Ind Med. (2004) 46:637–48. 10.1002/ajim.20106 15551378

[13] Kelly G Gana P Nielsen T MacGregor F. The incidence of potential conjunctival contamination in tonsillectomy. J R Coll Surg Edinb. (2000) 45:288–90. 11077775

[14] Marasco S Woods S. The risk of eye splash injuries in surgery. Aust N Z J Surg. (1998) 68:785–7. 10.1111/j.1445-2197.1998.tb04677.x 9814742

[15] Endo S Kanemitsu K Ishii H Narita M Nemoto T Yaginuma G. Risk of facial splashes in four major surgical specialties in a multicentre study. J Hosp Infect. (2007) 67:56–61. 10.1016/j.jhin.2007.05.020 17669549

[16] Hosoglu S Celen MK Akalin S Geyik MF Soyoral Y Kara IH. Transmission of hepatitis C by blood splash into conjunctiva in a nurse. Am J Infect Control. (2003) 31:502–4. 10.1016/j.ajic.2003.03.005 14647113

[17] Collins D Rice J Nicholson P Barry K. Quantification of facial contamination with blood during orthopaedic procedures. J Hosp Infect. (2000) 45:73–5. 10.1053/jhin.1999.0706 10833347

[18] Singh BI Nurein H Sinha S Singh S Housden P. Risk of conjunctival contamination in total joint arthroplasty. J Hosp Infect. (2006) 63:275–80. 10.1016/j.jhin.2006.01.037 16698118

[19] Keogh IJ Hone SW Colreavey M Walsh M. Blood splash and tonsillectomy: an underestimated hazard to the otolaryngologist. J Laryngol Otol. (2001) 115:455–6. 10.1258/0022215011908153 11429067

[20] Hansen D Krabs C Benner D Brauksiepe A Popp W. Laminar air flow provides high air quality in the operating field even during real operating conditions, but personal protection seems to be necessary in operations with tissue combustion. Int J Hyg Environ Health. (2005) 208:455–60. 10.1016/j.ijheh.2005.08.008 16325554

[21] Sadrizadeh S Pantelic J Sherman M Clark J Abouali O. Airborne particle dispersion to an operating room environment during sliding and hinged door opening. J Infect Public Health. (2018) 11:631–5. 10.1016/j.jiph.2018.02.007 29526441

[22] Noguchi C Koseki H Horiuchi H Yonekura A Tomita M Higuchi T. Factors contributing to airborne particle dispersal in the operating room. BMC Surg. (2017) 17:78. 10.1186/s12893-017-0275-1 28683726 PMC5500993

[23] Sunagawa S Koseki H Noguchi C Yonekura A Matsumura U Watanabe K. Airborne particle dispersion around the feet of surgical staff while walking in and out of a bio-clean operating theatre. J Hosp Infect. (2020) 106:318–24. 10.1016/j.jhin.2020.07.016 32702464

[24] Jagger J Hunt EH Brand-Elnaggar J Pearson RD. Rates of needle-stick injury caused by various devices in a University hospital. N Engl J Med. (1988) 319:284–8. 10.1056/NEJM198808043190506 3393183

[25] Centers for Disease Control and Prevention. Guidelines for prevention of transmission of human immunodeficiency virus and hepatitis B virus to health-care and public safety workers. MMWr Morb Mortal Wkly Rep. (1989) 38:S63–87. Available online at: https://www.cdc.gov/mmwr/preview/mmwrhtml/00001450.htm.

[26] International Health Care Worker Safety Center. EPI-Net: Uniform Blood and Body Fluid Exposure Report. (2003). Charlottesville, VA, University of Virginia Health System. Available online at: https://internationalsafetycenter.org/wp-content/uploads/reports/2003-Blood-and-Body-Fluid-Report.pdf. (accessed January 10, 2021).

[27] Centers for Disease Control and Prevention. National Hospital Discharge Survey: 2010 Table, Procedures by Selected Patient Characteristics. (2013). Retrieved from: http://www.cdc.gov/nchs/data/nhds/4procedures/2010pro4_numberprocedureage.pdf. (accessed January 4, 2021).

[28] Kurtz S Ong K Lau E Mowat F Halpern M. Projections of primary and revision hip and knee arthroplasty in the United States from 2005 to 2030. J Bone Joint Surg. (2007) 89:780–5. 10.2106/00004623-200704000-00012 17403800

[29] Iudicello S Fadda A. A road map to a comprehensive regulation on ventilation technology for operating rooms. Infect Control Hosp Epidemiol. (2013) 34:858–60. 10.1086/671261 23838232

[30] McHugh S Hill A Humphreys H. Laminar airflow and the prevention of surgical site infection. More harm than good? Surgeon. (2015) 13:52–8. 10.1016/j.surge.2014.10.003 25453272

[31] Cristina ML Spagnolo AM Sartini M Panatto D Gasparini R Orlando P. Can particulate air sampling predict microbial load in operating theatres for arthroplasty? PLoS ONE. (2012) 7:e52809. 10.1371/journal.pone.0052809 23285189 PMC3528722

[32] Pasquarella C Balocco C Colucci ME Saccani E Paroni S Albertini L. The influence of surgical staff behavior on air quality in a conventionally ventilated operating theatre during a simulated arthroplasty: a case study at the University Hospital of Parma. Int J Environ Res Public Health. (2020) 17:452. 10.3390/ijerph17020452 31936699 PMC7013425

[33] Wong KY Kamar HM Kamsah N Zawawi FM Tan H Musa MN. Correlation between particulate matter and microbial counts in hospital operating rooms. Adv Environ Biol. (2018) 12:1–5. 10.22587/aeb.2018.12.10.134458973

